# Potential drivers and implications of a balanced breeding sex ratio in a small population of an imperiled species with environmental sex determination

**DOI:** 10.1002/ece3.70166

**Published:** 2024-09-02

**Authors:** Ian Silver‐Gorges, Brian M. Shamblin, Mason Ashford, Paityn Bower, Mariana M. P. B. Fuentes

**Affiliations:** ^1^ Department of Earth, Ocean, and Atmospheric Science Florida State University Tallahassee Florida USA; ^2^ Warnell School of Forestry and Natural Resources University of Georgia Athens Georgia USA

**Keywords:** conservation genetics, microsatellite, paternity, sea turtle

## Abstract

Small populations of imperiled species are susceptible to the negative consequences of skewed sex‐ratios. In imperiled species with environmental sex determination such as sea turtles, examining sex ratios across a range of environments and population abundance levels can provide insight into factors that influence population resilience, which can then be the foci of management plans for these species. Breeding sex ratios (the ratio of actively breeding males to females during a reproductive season; BSRs) extrapolated from genetic parentage analyses are a common approach for enumerating sex ratios in sea turtles. Such analyses also allow for the characterization of multiple paternity within sea turtle clutches, which should reflect BSRs and breeding behaviors. We characterized the first BSR for a breeding assemblage of loggerhead sea turtles (*Caretta caretta*) belonging to the temperate, low‐abundance Northern Gulf of Mexico Recovery Unit using genotypes of 16 microsatellite loci from nesting females and hatchlings. Unlike prior studies at both more‐tropical and more‐temperate, and higher‐abundance, Recovery Units in this region, we found a balanced BSR of 1.3:1 males:female and a low incidence (~17%) of multiple paternity. This suggests that there are relatively few males breeding at this assemblage and within this Recovery Unit. Beaches in this region are expected to produce substantial numbers of male hatchlings based on sand temperature data. The relative dearth of mature males may then be due to hydrologic disturbances that disproportionately affect the fitness and survival of male hatchlings, or due to demographic stochasticity. More work is needed to study the factors that might influence male hatchling production and fitness in this region, particularly as climate change is predicted to lead to feminization in global sea turtle populations. Our work demonstrates the broad utility of characterizing BSRs and other sex ratios across a range of populations in imperiled, environmentally sensitive species.

## Graphical abstract

We characterized the first breeding sex ratio (BSR) for a breeding assemblage of loggerhead sea turtles (Caretta caretta) belonging to the temperate, low‐abundance Northern Gulf of Mexico Recovery Unit using genotypes of 16 microsatellite loci from nesting females and hatchlings. Unlike prior studies at both more‐tropical and more‐temperate, and higher‐abundance, Recovery Units in this region, we found a balanced BSR of 1.3:1 males:female and a low incidence (~17%) of multiple paternity. This suggests that there are relatively few males breeding at this assemblage and within this Recovery Unit.

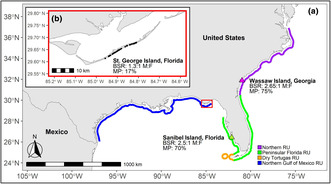

## 1 INTRODUCTION

Sex ratios are important demographic metrics that provide both proximal and evolutionary insight into key aspects of animal behavior (Székely et al., 2014). Primary sex ratios (PSRs; the ratio of sexually immature males and females) reflect unique evolutionary constraints (e.g., optimizing the production of male and female offspring when raising either sex incurs different costs; Clutton‐Brock et al., 1981; Oddie, 2000), or the relative costs and benefits of evolving gametic versus environmentally determined sex (Warner & Shine, 2008). Sex ratios of mature individuals include operational sex ratios (OSRs; the ratio of all sexually mature males and females) and breeding sex ratios (BSRs; the ratio of successful, actively breeding males and females). These ratios may differ from PSRs due to numerous ecological and behavioral factors present throughout ontogeny (Székely et al., 2014). OSRs and BSRs have direct behavioral and population dynamics relevance, as certain mating systems and breeding behaviors, such as sexually dichotomous breeding strategies or breeding periodicity, may lead to differences between OSRs and BSRs (Hager et al., 2020; Hays et al., 2010). OSRs and BSRs also influence competition for mates, mating systems, and reproductive output, typically via variations in female abundance (Breitwisch, 1989; Willson & Pianka, 1963).

Insight into breeding behaviors and population dynamics, which may be derived from the quantification of sex ratios, is important to inform the effective conservation of imperiled species (Kahn et al., 2021). In small or threatened populations, elongated periods of skewed (i.e., male‐ or female‐biased) OSRs or BSRs may lead to an increased risk of extinction via reduced reproductive output (Browne & Hecnar, 2007; Hays et al., 2010) and/or reduced genetic diversity (Chiba et al., 2023; Reid & Peery, 2014). Skewed sex ratios and associated negative impacts have been observed across a broad diversity of taxa, including but not limited to crustaceans (Chiba et al., 2023; Jury et al., 2019), fishes (Morgan & Trippel, 1996; Wilderbuer & Turnock, 2009), reptiles (Hays et al., 2017; Le Galliard et al., 2005), and birds (Homolková et al., 2024). These skewed ratios may be linked to sex‐specific mortality (Aresco, 2005; Corlatti et al., 2019; Székely et al., 2014; Wilderbuer & Turnock, 2009) or due to the impacts of dynamic environmental processes (e.g., El Niño‐Southern Oscillation, anthropogenic climate change) on resource availability (Székely et al., 2014; Williams et al., 2017). Anthropogenic climate change is also expected to drive changes in PSRs in species with environmentally determined sex (including most reptiles and some fishes), with numerous downstream implications for OSRs and BSRs, and subsequently for population dynamics and persistence (Pen et al., 2010). Due to the imminent, if not already ongoing, nature of perturbations to population dynamics, it is essential that baseline sex ratios be characterized for populations of imperiled, environmentally sensitive species (Donald, 2007; Robertson et al., 2006). Conservation efforts can then monitor changes in sex ratios over time or relate sex ratios to measures of reproductive output to assess how demographic structure influences population resilience, and ultimately design interventions to maintain demographic stability (i.e., maintain sex ratios at levels that ensure continued reproductive output: Robertson et al., 2006; Wedekind, 2012).

Characterizing baseline sex ratios is particularly important to increase understanding and guide successful conservation efforts in long‐lived, highly migratory, imperiled species, yet doing so is inherently challenging (Kahn et al., 2021). Sexually mature individuals of these species are difficult to empirically observe due to their vagility and time spent dispersed at foraging habitats, which has limited efforts to characterize OSRs (Covino et al., 2017; Gherardi‐Fuentes et al., 2020; Hays et al., 2010). Characterizations of BSRs may be relatively feasible as individuals return periodically to specific reproductive habitats where they may be enumerated (Kahn et al., 2021). Neonates are typically less vagile than adults, and there have been numerous efforts to characterize PSRs in long‐lived, highly migratory, imperiled species (Donald, 2007; Hays et al., 2022). However, in species with sexually monomorphic neonates, characterizing PSRs may require invasive, potentially destructive sampling or reliance upon estimations subject to broad variance (Ancona et al., 2017; Fuentes et al., 2017; Laloë et al., 2024). Overcoming these challenges to characterize baseline sex ratios in vulnerable populations of these species is a pressing conservation need (Fuentes et al., 2023; Kahn et al., 2021; Laloë et al., 2024). Interventions to maintain demographic stability (many of which are subject to debate regarding their ethics and efficacy; Fuentes et al., 2023; Patrício et al., 2021; Wedekind, 2012) need to be considered expediently, as generation times as long as 50 years in some species (e.g., bowhead whales, *Balaena mysticetus*; sea turtles, superfamily *Chelonioidea*; albatrosses, family *Diomedeidae*) mean that it will take time to observe conservation dividends (Fuentes et al., 2023; George et al., 2011; Heppell et al., 1999; Jouventin & Dobson, 2002; NMFS & USFWS, 2008). Without knowledge of baseline sex ratios, it is difficult to design effective conservation interventions towards this end (Donald, 2007; Fuentes et al., 2023; Mrosovsky & Yntema, 1980; Santidrián Tomillo et al., 2015; Wedekind, 2012).

Efforts to characterize baseline sex ratios (e.g., PSRs, OSRs, and BSRs) are ongoing in populations of all seven imperiled sea turtle species (Laloë et al., 2024). Populations of sea turtles globally have undergone historic declines and, in some cases, recent recovery (Mazaris et al., 2017). Sea turtles also exhibit environmental sex determination during embryonic development on sandy beaches (Mrosovsky & Yntema, 1980). Both of these facets of sea turtle biology and conservation may have altered or are currently altering sex ratios in sea turtle populations. OSRs and BSRs may be impacted by the same threats that lead to fluctuations in population abundance (although sex‐specific sources of mortality are largely unknown; Fuentes et al., 2023), and PSRs are highly susceptible to the impacts of anthropogenic climate change on nesting habitats (which may subsequently lead to altered OSRs and BSRs; Fuentes et al., 2011; Laloë et al., 2024). Work to characterize sex ratios in sea turtles has largely focused on estimating PSRs using nest temperature data as sea turtles are not sexually dimorphic until reaching sexual maturity, and have shown that PSRs are largely female biased (Laloë et al., 2024; Patrício et al., 2021). Furthermore, neonate sea turtles disperse vast distances from their natal beaches and spend 10–30 years developing prior to reaching sexual maturity (Avens et al., 2015; Bolten et al., 2003). Mature sea turtles spend much of their lives at broadly distributed foraging grounds where individuals from multiple populations overlap, and only undertake periodic breeding migrations to the vicinity of their natal beaches (Bowen & Karl, 2007; McClellan & Read, 2007; Schofield et al., 2010). Estimating OSRs is infeasible given sea turtle dispersal outside of reproduction, but BSRs have been estimated for some breeding populations (Patrício et al., 2021). BSRs have consistently demonstrated male biases, and many studies have simultaneously revealed multiple paternity in sea turtle nests, both of which suggest a substantial presence of mature male sea turtles (Hays et al., 2010; Lasala et al., 2013, 2018; Lee et al., 2018; Phillips et al., 2013; Schofield et al., 2017; Wright et al., 2012). However, it remains unclear exactly how shifts between PSRs and OSRs/BSRs occur; whether increasingly female‐biased PSRs will influence breeding behaviors and sex ratios of mature individuals; how BSRs vary latitudinally across a species' range (as we would expect less‐female biased PSRs, and potentially BSRs, in more temperate populations); how BSRs might vary with population abundance; or the threshold sex ratios at which population resilience begins to be impacted (Hays et al., 2022; Patrício et al., 2021). Additional BSR characterizations from previously unstudied populations facilitate comparisons between populations at different latitudes (and subsequently with different environmental characteristics) and with different abundances. These comparisons are needed to better understand sea turtle reproductive behaviors, and to aid conservation efforts in identifying the different environmental and anthropogenic factors that influence sex ratios across life stages, and ultimately influence demographic stability in populations of imperiled sea turtles.

**FIGURE 1:**
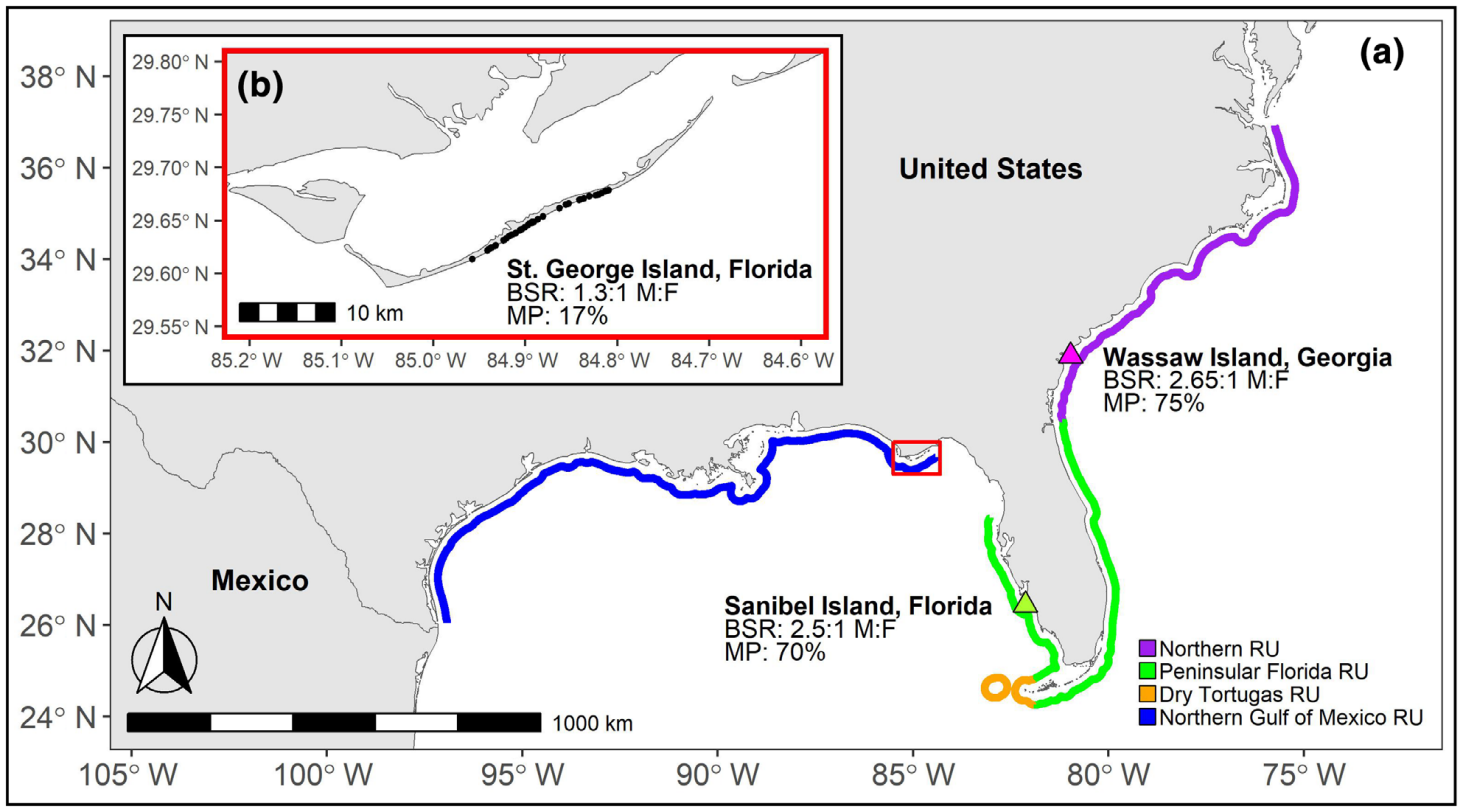
Panel a: Map of observed breeding sex ratios (BSRs) and occurrence of multiple paternity (MP) in the northwest Atlantic Ocean, and the extent of nesting beaches used by the different regional loggerhead Recovery Units (RUs). Findings from Wassaw Island and Sanibel Island reported in (Lasala et al., 2013, 2018). Panel b: Inset (red outline) shows BSR and MP for St. George Island and location of nests (black dots) sampled in the present study.

To this end, we sought to characterize the first BSR for loggerhead sea turtles (*Caretta caretta*) belonging to a low‐abundance, genetically discrete subpopulation in the temperate northern Gulf of Mexico. Loggerhead sea turtles nest in high densities along the southeast and Gulf coast of the United States (Ceriani et al., 2019; NMFS & USFWS, 2008). BSRs have only been quantified for assemblages within two subpopulations within this global Distinct Population Segment (Peninsular Florida Recovery Unit, Northern Recovery Unit) and were found to be male‐biased (2.65:1 males:females; Lasala et al., 2013, 2018). Loggerheads belonging to the Northern Gulf of Mexico Recovery Unit (NGM RU) nest on beaches that span from the western edge of Florida's Big Bend to the United States‐Mexico border (NMFS & USFWS, 2008; Shamblin et al., 2011, 2012). This population is estimated to comprise just ~880 nesting females (Ceriani et al., 2019), making it potentially susceptible to the negative impacts of demographic perturbations (Hays et al., 2010; Silver‐Gorges, Ceriani, et al., 2021). Breeding individuals in the NGM RU also utilize the northernmost, coolest, and therefore most male‐producing breeding habitats available in the Gulf of Mexico, which might be increasingly important to population resilience as climate change impacts begin to manifest in this region (Lamont et al., 2020; Montero et al., 2018). It is important to have an adequate understanding of regional variability in sex ratios to better constrain factors that influence demographic parameters and population resilience of loggerhead turtles in this region, including climate change impacts, habitat characteristics, mating behaviors, and threat exposure (Fuentes et al., 2020; Montero et al., 2018; Witt et al., 2010). To contribute to baseline knowledge of loggerhead sex ratios in the Gulf of Mexico, we sampled nesting females and hatchlings at St. George Island, Florida (Figure 1), the most abundant breeding assemblage in the NGM RU (Silver‐Gorges, Ceriani, et al., 2021), and used genetic parentage reconstruction to characterize the first BSR and rate of multiple paternity for the NGM RU. This baseline information will be informative to our understanding of sea turtle breeding dynamics, and to regional conservation efforts considering historic population fluctuations, as well as ongoing threats to loggerhead turtles across life stages in this region.

## 2 METHODS

### 2.1 Study site

St. George Island, Florida (Figure 1) hosts the most abundant nesting assemblage for loggerhead sea turtles in the NGM RU and provides habitat for approximately 400 loggerhead nests per year (Silver‐Gorges, Ceriani, et al., 2021). The island spans 33 km, and the beach is bifurcated into a 15.2‐km state park in the east, and a 17.8‐km public beach in the west. Nesting on St. George Island is most abundant in the public sector (Silver‐Gorges, Ceriani, et al., 2021), which is where sampling effort was focused.

### 2.2 Nesting female surveys

Surveys to collect tissue samples from nesting loggerheads and to mark nests for hatchling sampling occurred during the approximate peak of the 2022 nesting season at St. George Island, from June 19 to July 3 (Silver‐Gorges, Ingels, et al., 2021). This maximized encounters with females at various stages of their breeding seasons and was designed to mitigate sampling females with depleted sperm stores (i.e., those that began nesting earlier in the season) which could lead to the underrepresentation of sires (Lasala et al., 2020). Any encountered females were engaged following oviposition, or while returning to the water following a non‐nesting emergence (false crawl). Technicians checked for flipper and PIT identification tags and applied these tags when necessary for individual identification. Technicians then used 5 mm biopsy punches to collect epidermis samples from the shoulders of encountered females, which were stored in 95% EtOH until DNA extraction. The public beach at St. George Island is divided into approximately 2.4‐km‐long sections labeled A–J from east to west to aid morning surveys for sea turtle nests (Silver‐Gorges, Ceriani, et al., 2021). All clutches laid during the survey period were given a unique ID based on their section and the ascending numerical order in which they were laid within each section throughout the nesting season (e.g., A1 is the first clutch deposited in section A for the season).

### 2.3 Hatchling sampling

Nests laid during the survey period were caged 45 days after oviposition to retain hatchlings for sampling. Cages measured 0.6 × 0.6 × 0.6 m and were constructed out of 0.5″ mesh hardware cloth pursuant to Florida Fish and Wildlife Conservation Commission (FFWCC) guidelines (FFWCC, 2016). Caged nests were monitored three times per evening for emerging hatchlings. Upon emergence, all hatchlings above the surface were restrained with the goal of collecting tissue samples from 20 hatchlings (Lasala et al., 2018). The rear margin of the front flipper was sampled using a 1‐mm biopsy punch, and samples were stored in 95% EtOH until DNA extraction. All hatchlings were released together following sampling to minimize mortality during dispersal from the beach. Samples were collected from any dead hatchlings or late‐stage embryos remaining in the nest during nest productivity assessments conducted 3 days following hatchling emergence.

### 2.4 Genetic analyses

Parentage reconstruction techniques require genetic data from siblings at a minimum to infer both paternal and maternal genotypes (Jones & Wang, 2010). Single‐parent inferences (i.e., paternity or maternity analyses) often have higher confidence when one parent is known (Wang & Santure, 2009). Parentage inference when no parents are known can be accurate if the loci used have adequate allelic richness (Isberg, 2022), and we therefore caged and sampled all nests laid during the survey period, even if we had not encountered and sampled the female that had laid a given clutch.

All samples were shipped to the Shamblin Lab at the University of Georgia's Warnell School of Forestry for DNA extraction and microsatellite genotyping. Genomic DNA was extracted in 96‐well plate format. After evaporating off EtOH in a hood overnight, 50 μL of 10% Chelex‐100 (Sigma‐Aldrich) solution was added to each sample (Walsh et al., 1991), and each plate was heated on a thermal cycle for 20 min at 99.9°C to extract DNA. Each individual was genotyped at 16 highly polymorphic microsatellite loci isolated from loggerhead sea turtles (Shamblin et al., 2007, 2009). We conducted PCR amplifications in three 10 μL multiplex reactions as previously described (Shamblin et al., 2017) using 1 μL of DNA extract per reaction. Fragment analysis was conducted at Cornell University's Institute of Biotechnology on a 3730xl DNA Analyzer (Applied Biosystems) using GeneScan LIZ‐500 size standard. Negative controls were included in each DNA extraction and PCR plate to detect reagent contamination. Microsatellite diversity statistics and exclusion probabilities (i.e., the probabilities of failing to exclude unrelated individuals as parents with data from neither or one known parent) were calculated using Cervus v.3.0.7 (Kalinowski et al., 2007).

### 2.5 Breeding sex ratio

To estimate the BSR of loggerhead turtles at St. George Island, we implemented parentage reconstruction in COLONY 2.0 (Jones & Wang, 2010). Individuals were only included in parentage analysis if they were missing data at no more than three loci (≤~18.8% missingness). COLONY was run twice; first to estimate marker error rates, and then to infer parentage. In each case, COLONY was run for five “Very Long” runs of the Full‐Likelihood parentage inference method, which is the most computationally intensive but most accurate method employed by COLONY (Wang, 2012). Inbreeding and polygamy were allowed in these runs, and there was no sibship scaling or size priors. COLONY was allowed to update allele frequencies. In addition to hatchling and female genotypes, COLONY was also provided with known sibships (i.e., hatchlings sampled from the same nest or from nests laid by the same female). In the first COLONY run, marker error rates were assumed to be 0.001. Updated estimated marker error rates from this first run were then used to inform the second COLONY run. All relationships were manually assessed for accuracy. Additionally, electropherograms for offspring representing any singleton inferred males (i.e., males that sired one hatchling in a nest) were manually reexamined for accuracy. Following checking, the numbers of inferred males and females (both observed and inferred), were used to calculate the BSR. We report BSR based on all clutches, clutches with ≥10 sampled and genotyped hatchlings, and clutches with ≥15 sampled and genotyped hatchlings. This approach also allowed us to characterize multiple paternity in clutches laid at St. George Island, which we report under the same scheme as BSR (i.e., for all clutches, for clutches with ≥10 hatchlings, and for clutches with ≥15 hatchlings).

## 3 RESULTS

### 3.1 Female and hatchling sampling

During the June 2022 survey period, 29 females were encountered and sampled. Of these, 23 had nested and six had false crawled. In addition to the 23 nests representing sampled females, 24 nests were dug by unencountered females during the survey period. Thus, a total of 47 nests were caged for hatchling sampling (Figure 1). A total of 658 hatchlings were sampled from 40 of these nests (mean = 17 ± 6 SD hatchlings per nest; min = 1 hatchling; max = 22 hatchlings). Hatchlings did not develop (likely due to inundation) in seven caged nests that were not sampled.

### 3.2 Genetic analyses

Amplification was successful in 653 samples (29 females, 624 hatchlings from 39 nests). Thirty‐four samples, including samples from an entire nest, failed to amplify at three or more loci and were excluded from analyses. One clutch (*n* = 20 hatchlings) represented a hybridization event between a female loggerhead and one male green sea turtle (*Chelonia mydas*; suspected based on hatchling morphology and confirmed in microsatellite data, B.S. pers. comm.) and was also excluded from downstream analyses. Microsatellite diversity statistics from remaining samples are included in Table S1. Overall exclusion probabilities for all 16 loci with data from neither or one known parent were both <9 × 10^−8^, indicating that analyzing data from all loci would produce accurate parentage reconstructions.

### 3.3 Breeding sex ratio

Parentage reconstruction analyses included 632 individuals (29 females, 604 hatchlings from 38 nests). COLONY inferred parentage of hatchlings by 43 males and 33 females (10 inferred females, 23 known females) in all clutches in our sample (Table 1). All inferences, including four singleton males (#'s 19, 24, 37, 39), were manually verified to be accurate (Table 1). The estimated BSR of turtles breeding at St. George Island was 1.3:1 males:females for all nests (*n* = 38), 1.35:1 males:females for nests with ≥10 sampled hatchlings (*n* = 32), and 1.35:1 males:females for nests with ≥15 sampled hatchlings (*n* = 29).

**TABLE 1:** Observed and inferred parentage, likelihood of inferred parentage, and sample size for analyzed nests.

Nest ID	Mother	Father(s)	Likelihood	Hatchlings sampled
A16	KKM0976	1	1	22
A17	KKM0949	2	1	9
A18	#1	3	1	19
B10	#2	4, 5	0.9561	19
B11	KKM0925	6, 7, 8, 9, 10	0.0179	18
B12	#3	11	1	20
B13	KKM0967	12	1	20
B14	KKM0976	1	1	17
C10	#4	13	1	20
C7	KKM0967	12	1	18
C8	KKM0918	14	0.9347	4
CC5	#5	15	1	17
CC6	KKM0954	16	1	19
D12	#6	17, 18	0.9606	20
F15	KKM0932	19, 20	0.953	19
F18	KKM0956	21	1	18
F19	KKM0958	22	1	5
F20	KKF020	23	1	20
F21	KKF004	24	0.6586	1
F22	KKM0929	25	1	20
G25	MML099	26	0.9984	15
G26	KKF043	27	1	17
G27	KKF010	28	1	19
G28	#7	29	1	11
G29	KKM0983	30	1	20
G31	KKM0918	14, 31	0.9347	10
H18	KKM0983	30	1	18
H19	KKM0930	32	1	20
H21	KKF063	33	1	10
H22	KKM0965	34	0.524	20
H24	KKM0984	35	1	19
I18	#8	36, 37, 38	0.8008	20
I19	KKM0929	25	1	5
I20	KKM0927	39	0.9998	1
I22	LLT759	40	1	16
I23	KKM0973	41	1	20
J16	#9	42	1	19
J17	#10	43	1	19

*Note*: Mothers with “#” IDs are inferred, as are all fathers.

### 3.4 Paternity and breeding dynamics

**FIGURE 2:**
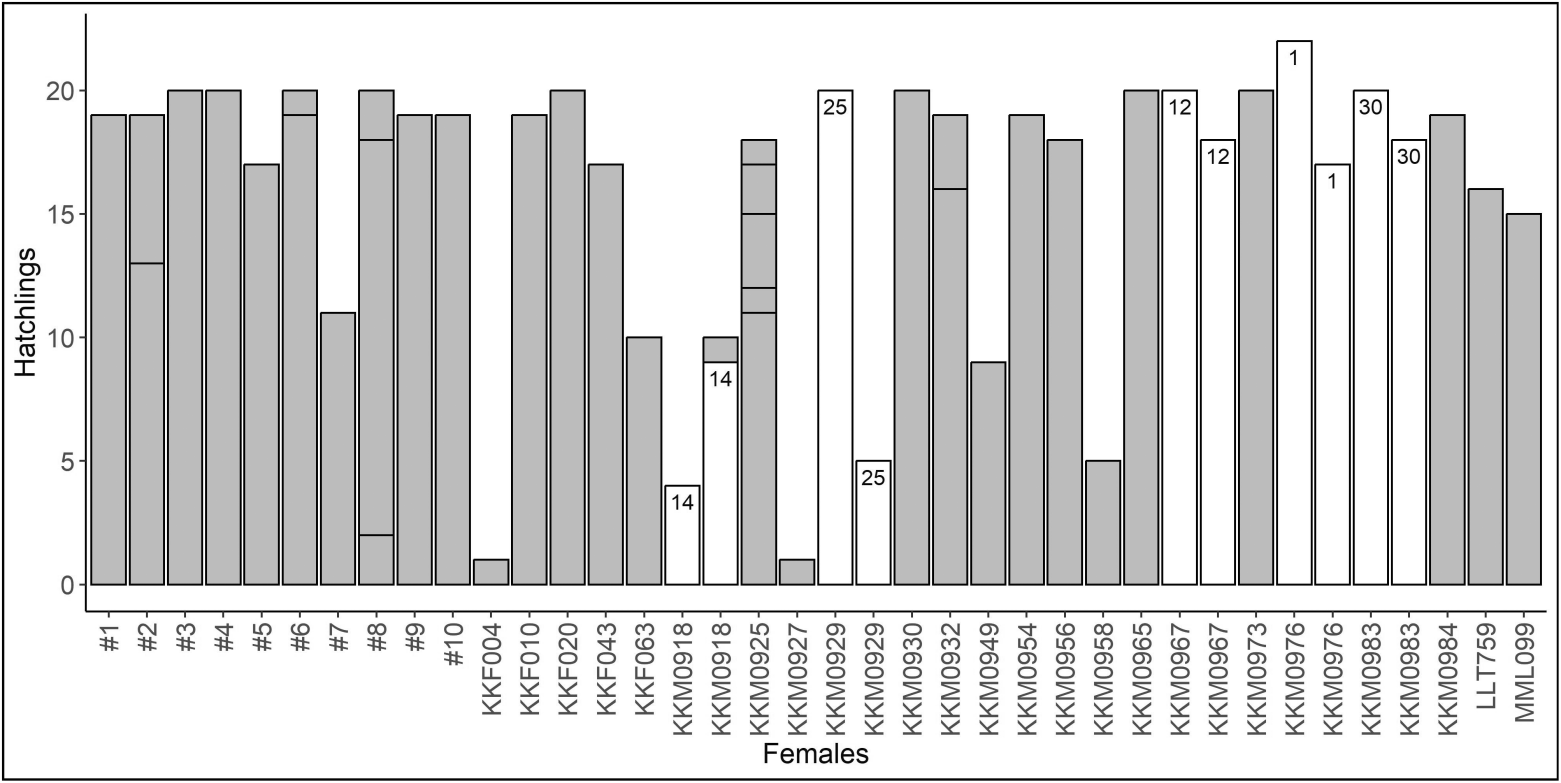
Number of hatchlings sired by different males in sampled nests (individual bars), identified by nesting female. Breaks within bars indicate different males. White bars indicate hatchlings sired by repeat males (i.e., males with offspring in multiple nests), and number indicates the unique male ID. For example, male # 1 sired every hatchling in both clutches laid by female KKM0976.

With all nests considered, 32 clutches had one sire, four clutches had two sires, one nest had three sires, and one nest had five sires (mean = 1.26 sires/nest), a rate of multiple paternity of 15.8%. All clutches with multiple paternity had ≥10 sampled hatchlings, and the rate of multiple paternity when considering only clutches with ≥10 sampled hatchlings was 18.8% (mean = 1.38 sires/nest). Only one nest with multiple paternity (G31, two sires; Table 1) had <15 sampled hatchlings. The rate of multiple paternity for clutches with ≥15 sampled hatchlings was 17.9% (mean = 1.32 sires/nest). In clutches with multiple paternity, one sire fathered the majority (>60%) of sampled hatchlings. These primary sires fathered an average of 78.9 ± 13.1% of sampled hatchlings in these clutches, while secondary sires fathered an average of 12.8 ± 8.0% of sampled hatchlings in these clutches. While five males were detected as sires in multiple clutches, there was no evidence of polygyny in these data. Further, of these repeat detections, only one was in a nest with multiple paternity (G31, Table 1; female KKM0918, Figure 2), and the repeat male sired 90% (*n* = 9) of the sampled hatchlings.

## 4 DISCUSSION

A relatively balanced (i.e., near 1:1 male to female) BSR, as well as a low occurrence of multiple paternity in sampled clutches, were found for the loggerhead turtle breeding assemblage at St. George Island. This may be representative of the breeding dynamics for the NGM RU, as St. George Island is the largest assemblage in this population (Silver‐Gorges, Ceriani, et al., 2021). Balanced or even male‐biased BSRs are thought to be due to short (i.e., 1 year) mating remigration intervals exhibited by male loggerhead turtles that counteract any female biases in OSRs (Hays et al., 2010; Lasala et al., 2018) and may also lead to high incidences of multiple paternity (Lee et al., 2018). However, the relatively balanced BSR and low incidence of multiple paternity at St. George Island are not typical for loggerhead populations in the northwest Atlantic Ocean. In the Northern Recovery Unit (specifically at Wassaw Island, Georgia), the BSR was found to be 2.65 males:females, the rate of multiple paternity was 75%, and there were an average of 2.65 sires/nest (Lasala et al., 2013). Similar observations were made in the Peninsular Florida Recovery Unit, specifically at Sanibel Island, Florida (Lasala et al., 2018). There are no other explicit molecular studies of BSRs for loggerhead sea turtles, but studies of multiple paternity in loggerhead breeding in Western Australia (Tedeschi et al., 2015), Japan (Sakaoka et al., 2011), and Greece (Zbinden et al., 2007) indicate high rates of multiple paternity and subsequently a male‐biased sex ratio in their samples. However, these studies utilized anywhere from two to four microsatellite loci for their analyses (the present study utilized 16), and it is possible that their findings would differ if additional loci were used (Isberg, 2022). A balanced sex ratio was observed for a loggerhead breeding assemblage in Greece (Hays et al., 2010), but the results of that study are likely not comparable to those presented here due to vast methodological differences (i.e., visual observations of males and females in water near a nesting beach over 27 days). Thus, the results of this study are best compared to those from other Recovery Units within the same region.

The differences in BSRs and paternity between the NGM RU and other Recovery Units in the northwest Atlantic Ocean may be due to environmental effects. Incubation conditions, specifically temperature, influence PSRs which may subsequently influence OSRs within Recovery Units (Fuentes et al., 2011; Laloë et al., 2024; Mrosovsky & Yntema, 1980). Male turtles are produced at cooler temperatures, while females are produced at warmer temperatures (Mrosovsky & Yntema, 1980). The breeding habitat throughout the NGM RU lies at a near intermediate latitude between breeding habitats within the Northern and Peninsular Florida Recovery Units, and also provides incubation environments that should produce a high proportion of male hatchlings (Lamont et al., 2020; Montero et al., 2018). At St. George Island specifically, the average incubation temperature of a subset of nests (*n* = 17) laid during the survey period for this study was 29.8 ± 1.3°C (ISG and MF, pers. comm.), in line with measurements from other beaches within the NGM RU (Lamont et al., 2020; Montero et al., 2018). Based on measured incubation temperatures in this region, and how incubation temperatures tend to decrease with increasing latitude (Wyneken & Lolavar, 2015), we expect beaches within the Northern Recovery Unit (e.g., Wassaw Island) to produce more male hatchlings, and beaches within the Peninsular Florida Recovery Unit (e.g., Sanibel Island) to produce fewer male hatchlings, than beaches within the NGM RU. There are numerous factors that may influence how PSRs transition to OSRs and BSRs (i.e., sex‐biased survival rates), but without evidence of sex‐specific sources of mortality, we would expect that the number of male adults within a population should scale to some extent with the number of male hatchlings produced at beaches used by those populations. This is the case for green sea turtles (*Chelonia mydas*) in eastern Australia, where populations in the more‐temperate south produce more male hatchlings and have a higher proportion of immature and mature males than populations in the more‐tropical north (Jensen et al., 2018). Assuming that loggerhead populations in the northwest Atlantic Ocean follow this dynamic, observed male‐biased BSRs likely reflect cooler incubation temperatures (within the Northern Recovery Unit, specifically) and how male breeding periodicity counteracts female biases in OSRs (potentially stemming from female‐biased PSRs) within more tropical populations (within the Peninsular Florida Recovery Unit, specifically; Hays et al., 2010; Lasala et al., 2013; Lolavar & Wyneken, 2015; Lasala et al., 2018).

It is possible that incubation environments within the NGM RU have influenced PSRs beyond influencing sex‐determination. Nesting beaches within the NGM RU are particularly vulnerable to hydrologic disturbances, such as nest inundation during high tides and tropical cyclones, and excessive precipitation (Brost et al., 2015; Fuentes et al., 2019; Montero et al., 2018; Silver‐Gorges, Ceriani, et al., 2021; Ware et al., 2021). Male hatchlings are produced at cooler temperatures, often presumed to be deeper in nests, in areas of the nest that are more susceptible to hydrologic disturbances and periodic inundation (Booth & Astill, 2001; Ware & Fuentes, 2018). Even if nesting beaches within the NGM RU should produce more male hatchlings than beaches within more tropical Recovery Units (e.g., the Peninsular Florida RU), these hatchlings may not survive, or be as fit, if they are impacted by hydrological disturbances (Gatto & Reina, 2020, 2022; Montero et al., 2018). Such a dynamic, which may be prevalent throughout the NGM RU, could reduce male turtle abundance, and lead to the relatively balanced BSR and low incidence of multiple paternity we observed at this assemblage.

If environmental conditions are not influencing BSRs and rates of multiple paternity in the NGM RU, it is possible that demographic stochasticity (i.e., random survival) influences male abundance within this and other Recovery Units. In small populations in particular, random sampling (i.e., survival) of individuals that make it to maturity might lead to anomalous BSRs (Le Galliard et al., 2005), and subsequently to anomalous observations of multiple paternity in sea turtles (Lee et al., 2018). The Northern and Peninsular Florida Recovery Units are two to 50 times more abundant, respectively, in terms of nests laid than the NGM RU, which likely reflects the relative abundance of breeding individuals (Ceriani et al., 2019; NMFS & USFWS, 2023; Silver‐Gorges, Ceriani, et al., 2021). Random sampling of individuals surviving to maturity and breeding in the lower‐abundance NGM RU could have given rise to the low BSR and rate of multiple paternity observed in loggerheads in this Recovery Unit. Conversely, populations may be large enough in the Northern and Peninsular Florida Recovery Units to buffer this random sampling, and as such BSRs and rates of multiple paternity are consistent between these Recovery Units.

Through characterizing the first BSR for a loggerhead breeding assemblage within the NGM RU, we elucidated previously unknown variability in BSRs between Recovery Units that highlights potential concerns for the suitability of the incubation environments for loggerhead turtles in this region. Sea turtle populations are predicted to undergo feminization as climate change progresses and beaches in the northwest Atlantic Ocean warm (Fuentes et al., 2011; Laloë et al., 2024; Patrício et al., 2021). Although male breeding periodicity and polyandry may maintain hatchling production within sea turtle Recovery Units, populations with already reduced male abundance such as the NGM RU may be the most vulnerable to potential negative impacts of reduced genetic diversity and ultimately to demographic collapse due to increasing male scarcity (Hays et al., 2010; Mitchell et al., 2010). Decreasing genetic diversity in these populations may lead immediately to declines in individual fitness parameters, and ultimately to reduced adaptive potential (Maurer et al., 2021). While evidence for this dynamic has yet to be empirically observed in small populations of sea turtles (Maurer et al., 2021), it has been observed in fishes (Vrijenhoek, 1994), crustaceans (Markert et al., 2010), and mammals (Furlan et al., 2012), among other taxa. However, it is important to note that polygyny is a prevalent dynamic in sea turtle breeding assemblages and populations when male abundance is critically low (Gaos et al., 2018). The NGM RU does not appear to have reached this point yet, and the lack of polygyny we observed (in ~14% of nests laid at St. George Island in 2022) is consistent across the NGM, Northern, and Peninsular Florida Recovery Units. Additionally, suspected male‐mediated gene flow between the NGM RU and Peninsular Florida Recovery Unit, which may be realized due to opportunistic mating events as NGM RU females cross Peninsular Florida Recovery Unit breeding areas while migrating from foraging to NGM RU breeding areas, could mitigate some expected negative impacts of reduced NGM RU male turtle abundance (Bowen et al., 2005; Hart et al., 2014).

Still, with potentially increasingly feminized hatchling production, it may only be a matter of time before the NGM RU crosses below a threshold abundance of mature male turtles, particularly if male abundance is further mitigated by expected increases in hydrologic disturbances to incubating clutches due to changes in climate in this region. Baseline characterizations of BSRs are critical for assessing the suitability of these habitats for loggerheads into the future. Additional research in the NGM and other Recovery Units should seek to quantify male hatchling production and sex ratios at multiple breeding assemblages, investigate potential differences in survival or fitness between male and female hatchlings, and determine if any incubation conditions (e.g., temperature and moisture) might be related to inter‐ or intra‐regional differences in male hatchling production or performance (Booth, 2017; Gatto & Reina, 2022). Such differential mortality, if it is occurring, could more easily engender the decline in genetic diversity and male scarcity (mentioned above) in small populations of sea turtles than in large populations (Maurer et al., 2021). The comparisons made here use data from individual breeding assemblages as proxies for entire Recovery Unit BSRs, and there is a need for additional characterizations of BSRs from other assemblages within these Recovery Units to confirm that findings from these individual assemblages are not anomalous. The information generated from holistic studies of sex ratios, fitness, and environmental differences could be used to inform updates to regional management plans (e.g., by highlighting habitats that may require protection and/or intervention) considering climate change. Such work was conducted over 3 decades in hatchling, immature, and adult green turtles in eastern Australia, and showed that feminized hatchling production was leading to increasingly feminized immature and mature turtles (Jensen et al., 2018). This specific study took advantage of a unique long‐term, multifaceted dataset, but subsequent efforts to holistically model how PSRs influence OSRs and BSRs within additional sea turtle populations would enable researchers and management agencies to make inferences about sex ratios across life stages. This could identify the specific factors that most influence variability in sex ratios within and among sea turtle populations based on limited data (e.g., confidently making qualitative inferences about PSRs based on BSRs). We encourage future research efforts to quantify sex ratios across life stages and to undertake our suggested future research directions in additional sea turtle populations, as well as population of other imperiled, environmentally sensitive species, for which sex ratios have yet to be holistically characterized.

## AUTHOR CONTRIBUTIONS

**Ian Silver‐Gorges:** Conceptualization (lead); data curation (lead); formal analysis (lead); funding acquisition (lead); investigation (lead); methodology (equal); project administration (supporting); visualization (lead); writing – original draft (lead); writing – review and editing (lead). **Brian M. Shamblin:** Conceptualization (supporting); data curation (equal); formal analysis (equal); funding acquisition (equal); investigation (equal); methodology (equal); project administration (supporting); resources (equal); validation (equal); visualization (supporting); writing – original draft (supporting); writing – review and editing (supporting). **Mason Ashford:** Data curation (supporting); investigation (supporting); methodology (supporting); writing – original draft (supporting); writing – review and editing (supporting). **Paityn Bower:** Data curation (supporting); investigation (supporting); methodology (supporting); writing – original draft (supporting); writing – review and editing (supporting). **Mariana M. P. B. Fuentes:** Conceptualization (equal); data curation (supporting); formal analysis (supporting); funding acquisition (equal); investigation (supporting); methodology (supporting); project administration (lead); resources (lead); supervision (lead); validation (equal); visualization (supporting); writing – original draft (supporting); writing – review and editing (supporting).

## CONFLICT OF INTEREST STATEMENT

The authors declare that they have no competing interests with the publication of the manuscript.

## Supporting information


Appendix S1

Data S1

## Data Availability

All raw microsatellite genotypes and R code used to generate statistics and figures are available on Dryad (https://doi.org/10.5061/dryad.cfxpnvxfx) and GitHub (https://github.com/isilverg/BSR_Analyses, https://github.com/FuentesLab/BSR_Analyses).
