# Supplementary material for: Potential drivers and implications of a balanced breeding sex ratio in a small population of an imperiled species with environmental sex determination
Source: Ecol Evol. 2024 Sep 2;14(9):e70166. doi: 10.1002/ece3.70166 (PMC11366973; doi:10.1002/ece3.70166)
Supplement: Supplementary file 2 — Data S1 [file ECE3-14-e70166-s002.docx]

**Appendix**

Appendix Table 1. Microsatellite diversity statistics calculated from hatchling data. k = number of alleles. N = number of individuals. H_O_ = observed heterozygosity. H_E_ = expected heterozygosity. PIC = polymorphic information content. NE-1P = non-exclusion probability for one candidate parent. NE-2P = non-exclusion probability for one candidate parent when the other parent is known. NE-PP = non-exclusion probability for a candidate parent pair. NE-I = non-exclusion probability for identity of two unrelated individuals. NE-SI = non-exclusion probability for identity of two siblings. HW = significance of deviation from Hardy-Weinberg equilibrium after Bonferroni correction (ND = not detected). Deviations from HWE were tested using one randomly selected hatchling genotype from each nest. F(Null) = estimated null allele frequency. Error = COLONY-estimated marker error rates.

| **Locus** | **k** | **N** | **H_O_** | **H_E_** | **PIC** | **NE-1P** | **NE-2P** | **NE-PP** | **NE-I** | **NE-SI** | **HW** | **F(Null)** | **Error** |  |
| --- | --- | --- | --- | --- | --- | --- | --- | --- | --- | --- | --- | --- | --- | --- |
| CcP2G10 | 22 | 604 | 0.945 | 0.922 | 0.915 | 0.275 | 0.16 | 0.042 | 0.012 | 0.292 | ND | -0.0133 | -0.0133 |  |
| CcP1H11 | 14 | 600 | 0.93 | 0.877 | 0.864 | 0.397 | 0.247 | 0.092 | 0.027 | 0.319 | ND | -0.0313 | -0.0313 |  |
| CcP1F01 | 13 | 603 | 0.897 | 0.875 | 0.862 | 0.403 | 0.251 | 0.095 | 0.028 | 0.32 | ND | -0.0124 | -0.0124 |  |
| CcP1G03 | 14 | 603 | 0.942 | 0.896 | 0.886 | 0.351 | 0.212 | 0.071 | 0.02 | 0.308 | ND | -0.0266 | -0.0266 |  |
| CCP1B03 | 18 | 603 | 0.882 | 0.872 | 0.859 | 0.406 | 0.254 | 0.095 | 0.029 | 0.322 | ND | -0.0058 | -0.0058 |  |
| CcP1G02 | 18 | 602 | 0.927 | 0.917 | 0.91 | 0.291 | 0.17 | 0.047 | 0.013 | 0.295 | ND | -0.0059 | -0.0059 |  |
| CcP5C08 | 14 | 604 | 0.954 | 0.898 | 0.888 | 0.345 | 0.208 | 0.069 | 0.02 | 0.306 | ND | -0.0312 | -0.0312 |  |
| CcP5H07 | 15 | 602 | 0.882 | 0.887 | 0.876 | 0.373 | 0.228 | 0.081 | 0.023 | 0.313 | ND | 0.0024 | 0.0024 |  |
| CcP5F01 | 29 | 603 | 0.942 | 0.936 | 0.932 | 0.231 | 0.131 | 0.029 | 0.008 | 0.284 | ND | -0.0046 | -0.0046 |  |
| CcP2H12 | 14 | 604 | 0.849 | 0.84 | 0.824 | 0.472 | 0.306 | 0.13 | 0.041 | 0.341 | ND | -0.0083 | -0.0083 |  |
| CcP7G11 | 12 | 603 | 0.874 | 0.864 | 0.849 | 0.429 | 0.272 | 0.109 | 0.033 | 0.327 | ND | -0.005 | -0.005 |  |
| CcP7C06 | 12 | 583 | 0.839 | 0.865 | 0.85 | 0.43 | 0.272 | 0.11 | 0.033 | 0.326 | ND | 0.0158 | 0.0158 |  |
| CcP7C04 | 13 | 604 | 0.954 | 0.903 | 0.894 | 0.332 | 0.198 | 0.063 | 0.018 | 0.303 | ND | -0.0288 | -0.0288 |  |
| CcP7B07 | 20 | 600 | 0.93 | 0.91 | 0.903 | 0.305 | 0.18 | 0.051 | 0.015 | 0.299 | ND | -0.0104 | -0.0104 |  |
| CcP7E11 | 11 | 603 | 0.841 | 0.842 | 0.822 | 0.484 | 0.316 | 0.144 | 0.044 | 0.341 | ND | -0.0002 | -0.0002 |  |
| CcP8E07 | 16 | 603 | 0.886 | 0.886 | 0.875 | 0.374 | 0.229 | 0.081 | 0.024 | 0.313 | ND | 0.0013 | 0.0013 |  |
